# Visits to Private Asylums

**Published:** 1903-01-24

**Authors:** 


### VISITS TO PRIVATE ASYLUMS.

By Our Rambling Reporter.

### COURT HALL, DEVONSHIRE.

This asylum is situated in the village of Kenton, and it may be reached by train from Exeter to Starcross or by steam ferry from Exmouth to Starcross. In either case a walk of about a mile and a half brings one to Kenton, and Court Hall is easily found in a quiet street of this quiet little village. On the way we get a glimpse of Powderham Castle, the seat of the Earl of Devon. The manor came to the family through Lady Margaret Bohun, granddaughter of Edward I. The parish church is passed on the right, and nearly opposite is the entrance to Court Hall, which is supposed to have been the dower house of Powderham, and in it Polwheele wrote his history of the county.

The hall is licensed for eight lady patients, and the license dates from 1868. It is the only private asylum in this part of the county, and on the occasion of our visit it contained its full number of residents, but two of them were voluntary boarders. One of these had been a patient in the asylum and had remained as a voluntary boarder for a year or more, a fact which speaks most highly for the manner in which the patients are treated and cared for. It would, indeed, be impossible to imagine anything further removed from the old popular idea of an asylum than is Court Hall. It is, in fact, a private house, pure and simple, without a single element of the institution about it, and it is the intention of the present proprietress to keep it so, a decision which we need hardly say is in perfect harmony with our own ideas of what these small asylums should be. Court Hall is a veritable home of rest for the wearied in brain, and here, if anywhere, recovery might be expected to take place in acute cases where recovery was possible, or, failing that, contentment of the invalid ought to ensue.

The patients' bedrooms are unusually good, both as regards their size and the nice, bright way in which they are furnished. Each lady has a room to herself, and some have provision for a nurse to sleep in the room should any circumstance arise making that precaution necessary or desirable.

The grounds surrounding the house are of four acres in extent, and the gardens are both secluded and cheerful. The payments range from £3 3s. a week up to £7 7s. or more, according to the accommodation required and the nature of the case. For instance, if special nurses had to be employed for any particular patient of course the higher rates would have to be charged.

